# Supplementary material for: A signal transmission strategy driven by gap-regulated exonuclease hydrolysis for hierarchical molecular networks
Source: Commun Biol. 2024 Mar 16;7:335. doi: 10.1038/s42003-024-06036-5 (PMC10944543; doi:10.1038/s42003-024-06036-5)
Supplement: Supplementary file 2 — Supplementary Information [file 42003_2024_6036_MOESM2_ESM.pdf]

A signal transmission strategy driven by gap-regulated exonuclease hydrolysis for hierarchical molecular networks

Xin Liu[1], Xun Zhang[1], Shuang Cui[1], Shujuan Xu[2], Rongming Liu[3], Bin Wang[4], Xiaopeng Wei[1], Qiang Zhang[1]*

[1] School of Computer Science and Technology, Dalian University of Technology, Dalian, Liaoning 116024, China.

[2] Key Lab of Biotechnology and Bioresources Utilization of Ministry of Education, College of Life Science, Dalian Minzu University, Dalian, Liaoning 116600, China.

[3] MOE Key Laboratory of Bio-Intelligent Manufacturing, School of Bioengineering, Dalian University of Technology, Dalian, Liaoning 116024, China.

[4] Key Laboratory of Advanced Design and Intelligent Computing, Ministry of Education, School of Software Engineering, Dalian University, Dalian, Liaoning 116622, China.

* Correspondence to Qiang Zhang, e-mail: zhangq@dlut.edu.cn

Table of Contents

Supplementary Methods

Supplementary Tables 1-2

Supplementary Figures 1-37

Supplementary Methods

Chemicals and Materials

All DNA oligonucleotides were purchased from Sangon Biotech. Co., Ltd. (Shanghai). Exo III and Exo λ were purchased from New England Biolabs, Inc. (Beijing). DNA oligonucleotides were stocked with deionized water (18.2 MΩ.cm) at 4°C. The DNA oligonucleotides without modification were purified by PAGE, and 5'-phosphorylated, 3'-phosphorothioate, and fluorophore- and quench-modified DNA oligonucleotides were purified by HPLC. The concentration of DNA oligonucleotides was quantified with a Nanodrop 2000/2000c (Waltham). The incubation buffer for the experiments was 1× TAE/Mg^{2+}/BSA buffer (pH = 8.0) consisting of Tris (40 mM), acetic acid (20 mM), EDTA·2Na (1 mM), $Mg(OAc)_2 \cdot 6H_2O$ (12.5 mM), and BSA (50 μg ml^{-1}). All DNA sequences are shown in the Supplementary Table 1 and Table 2.

Methods

Annealing for the substrates

All substrates that were not single-stranded structures were prepared in 1× TAE/Mg^{2+} buffer (pH = 8.0) without BSA for the same annealing procedure, which was set to be heated to 90°C (kept for 5 min), then steadily cooled to 85°C at 1°C min^{-1} in 5 min, and finally steadily cooled to 25°C at 0.5°C min^{-1} in 2 h. The freshly annealed substrates were kept at room temperature for the experiments.

Processing for the fluorescence data

All fluorescence curves and columns were calculated by averaging the three sets of the repeated fluorescence experiments. For the fluorescence data in the first three parts of the article, the third data measurement in the kinetic cycles was recorded as the baseline, which was subtracted by the data collected from each cycle during the processing of the fluorescence values to obtain the final curve or column figure. In the last part of the article, the third data measurement in the kinetic cycles was recorded as the initial value, and the fluorescence data was collected from each kinetic cycle as the real-time value. The

fluorescence signal was summed automatically by the Sum & Out module in Fig. 6d and converted to numerical output through the calibration curve in Supplementary Fig. 23d.

Supplementary Tables

Supplementary Table 1. DNA sequences of mechanism and strategy verification

DNA Oligos	Sequences (from 5' to 3')
A	AGTCACTCCATAACTTACTCC/**idSp**/TCAATGTATCATACTCTTAAC***T*T*T*T**
A1	AGTCACTCCATAACTTACTCC
A2	ATCAATGTATCATACTCTTAAC***T*T*T*T**
A2(PO_4)	PO_4-ATCAATGTATCATACTCTTAAC***T*T*T*T**
A2(AP)	/**idSp**/TCAATGTATCATACTCTTAAC***T*T*T*T**
B	GTTAAGAGTATGATACATTGATGGAGTAAGTTATGGAGTGACT***T*T*T*T**
B11	GGAGTAAGTTATGGAGTGACT***T*T*T*T**
B12	GAGTAAGTTATGGAGTGACT***T*T*T*T**
B13	AGTAAGTTATGGAGTGACT***T*T*T*T**
B14	GTAAGTTATGGAGTGACT***T*T*T*T**
B15	TAAGTTATGGAGTGACT***T*T*T*T**
B16	AAGTTATGGAGTGACT***T*T*T*T**
B21	GTTAAGAGTATGATACATTGA***T*T*T*T**
B22	GTTAAGAGTATGATACATTG***T*T*T*T**
B23	GTTAAGAGTATGATACATT***T*T*T*T**
B24(A)	GTTAAGAGTATGATACATA***T*T*T*T**
B24C	GTTAAGAGTATGATACATC***T*T*T*T**
B24G	GTTAAGAGTATGATACATG***T*T*T*T**
B25(A)	GTTAAGAGTATGATACAA***T*T*T*T**
B25C	GTTAAGAGTATGATACAC***T*T*T*T**
B25G	GTTAAGAGTATGATACAG***T*T*T*T**
B26(T)	GTTAAGAGTATGATACT***T*T*T*T**

B26C	GTTAAGAGTATGATACC***T*T*T*T**
B26G	GTTAAGAGTATGATACG***T*T*T*T**
F1	**BHQ1**-AGTCACTCCATAACTTAGTTATGGA/i6FAMdT/***T*T*T*T**
F2	**BHQ2**-GTATCATACTCTTAACTTAAGAGTAA/iROXdT/***T*T*T*T**

Supplementary Table 2. DNA sequences of molecular computing model

Substrates Name	Domains Name	Sequences (from 5' to 3')
P1	ab1	CCACTAACTCACATTTATGTGATCATACATTCATCAAGTAG* **T*T*T*T**
	a*	**PO_4**-CACATAAATGTGAGTTAGTGG***T*T*T*T**
	m1*b*	CATGTGATGTTGAGATATAGCTACTTGATGAATGTATGAT ***T*T*T*T**
W1	a*c	CACATAAATGTGAGTTAGTGG/**idSp**/TCAATGTATCATACTC TTAACTACAC***T*T*T*T**
	a1	AACTCACATTTATGTG***T*T*T*T**
	c*	GTGTAGTTAAGAGTATGATACAT***T*T*T*T**
1'	b2m1	CTATATCTCAACATCACATG***T*T*T*T**
P2	db1	CTCACAACTCACATTTATGTGATCATACATTCATCAAGTAG* **T*T*T*T**
	d*	**PO_4**-CACATAAATGTGAGTTGTGAG***T*T*T*T**
	m2*b*	AACTAAACTATGAGATATAGCTACTTGATGAATGTATGAT ***T*T*T*T**
W2	e*c	CACATAAATGTGAGTTGTGAG/**idSp**/TCAATGTATCATACTC TTAACTACAC***T*T*T*T**
	d1	AACTCACATTTATGTG***T*T*T*T**
	c*	GTGTAGTTAAGAGTATGATACAT***T*T*T*T**
2'	b2m2	CTATATCTCATAGTTTAGTT***T*T*T*T**
P3	eb1	AACGAGAAATGTCTTTAGACTATCATACATTCATCAAGTAG ***T*T*T*T**
	e*	**PO_4**-AGTCTAAAGACATTTCTCGTT***T*T*T*T**
	m3*b*	TTAGATTAGATGAGATATAGCTACTTGATGAATGTATGAT ***T*T*T*T**

W3	e*c	AGTCTAAAGACATTTCTCGTT/**idSp**/TCAATGTATCATACTC TTAACTACAC***T*T*T*T**
	e1	GAAATGTCTTTAGACT***T*T*T*T**
	c*	GTGTAGTTAAGAGTATGATACAT***T*T*T*T**
3’	b2m3	CTATATCTCATCTAA TCTAA***T*T*T*T**
P4	fb1	TATGGGAAGTGAGATTTCTCAATCATACATTCATCAAGTAG ***T*T*T*T**
	f*	**PO$_4$**-TGAGAAATCTCACTTCCCATA***T*T*T*T**
	m4*b*	TGTATACTAATGAGATATAGCTACTTGATGAATGTATGAT ***T*T*T*T**
W4	f*c	TGAGAAATCTCACTTCCCATA/**idSp**/TCAATGTATCATACTC TTAACTACAC***T*T*T*T**
	f1	GAAGTGAGATTTCTCA***T*T*T*T**
	c*	GTGTAGTTAAGAGTATGATACAT***T*T*T*T**
4’	b2m4	ATCATACATTCATCAAGTAGCTATATCTCA***T*T*T*T**
P5	gb1	ACAGGGAAGTGAGATTTCTCAATCATACATTCATCAAGTAG ***T*T*T*T**
	g*	**PO$_4$**-TGAGAAATCTCACTTCCCTGT***T*T*T*T**
	m5*b*	ATGATTAGTATGAGATATAGCTACTTGATGAATGTATGAT ***T*T*T*T**
W5	g*c	TGAGAAATCTCACTTCCCTGT/**idSp**/TCAATGTATCATACTC TTAACTACAC***T*T*T*T**
	g1	GAAGTGAGATTTCTCA***T*T*T*T**
	c*	GTGTAGTTAAGAGTATGATACAT***T*T*T*T**
5’	b2m5	CTATATCTCATACTAATCAT***T*T*T*T**
read	b	ATCATACATTCATCAAGTAGCTATATCTCA***T*T*T*T**
Rep	c1*	TTAAGAGTATGATACAT/i6FAMdT/***T*T*T*T**
	c	**BHQ1**-AATGTATCATACTCTTAACTACAC***T*T*T*T**

/idSp/: The apurinic/apyrimidinic (AP) site

*T: Phosphorothioate modified thymine base

PO_4: Phosphorylated modification

/i6FAMdT/: FAM modified thymine base

BHQ1: Black Hole Quencher 1 modification

/iROXdT/: ROX modified thymine base

BHQ2: Black Hole Quencher 2 modification

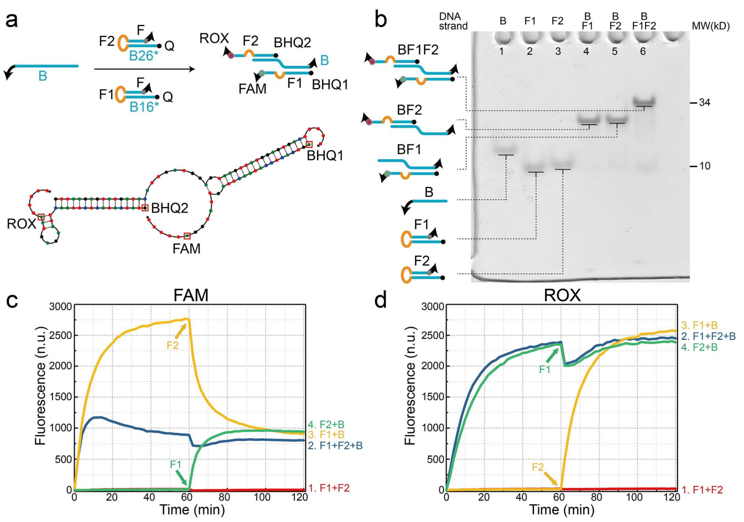

Supplementary Figure 1. The analysis of FAM fluorescence decrease. a, The reaction and structural simulation of BF1F2. **b,** The PAGE analysis of B opening F1 and F2. **c,** FAM fluorescence testing of B opening F1 and F2. **d,** ROX fluorescence testing of B opening F1 and F2.

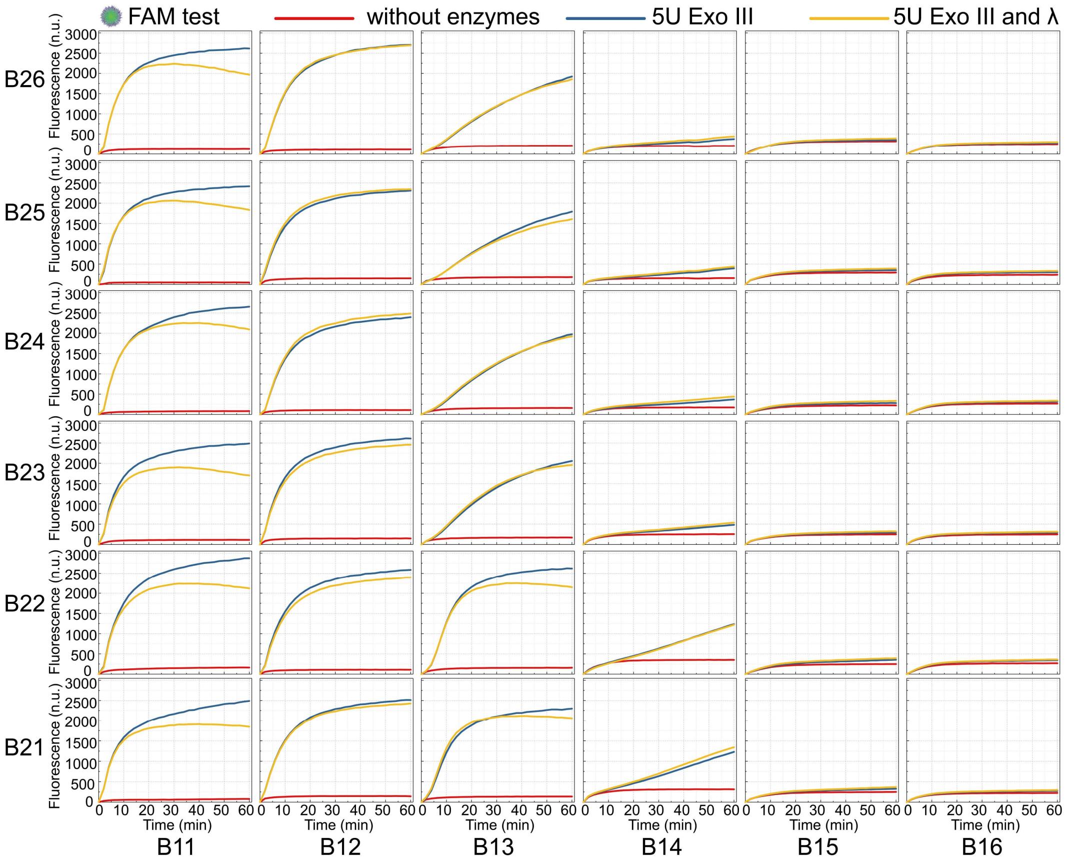

Supplementary Figure 2. FAM curves of the substrate with all gap combinations under different exonucleases. The hydrolysis of the substrates with all gap combinations is tested under different exonucleases backgrounds, which include a control group without enzymes, Exo III, and a coexistence of Exo III and Exo λ. The output signal B1 is monitored by F1.

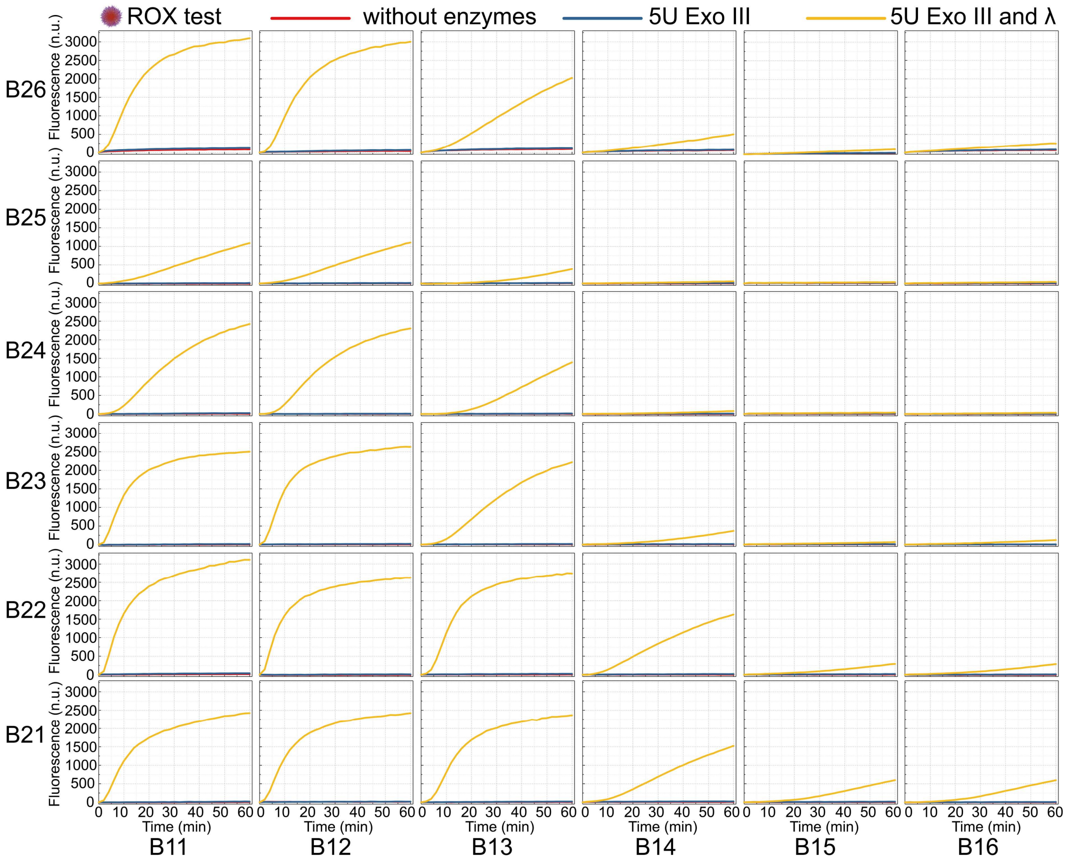

Supplementary Figure 3. ROX curves of the substrate with all gap combinations under different exonucleases. The hydrolysis of the substrates with all gap combinations is tested under different exonucleases backgrounds, which include a control group without enzymes, Exo III, and a coexistence of Exo III and Exo λ. The output signal B2 is monitored by F2.

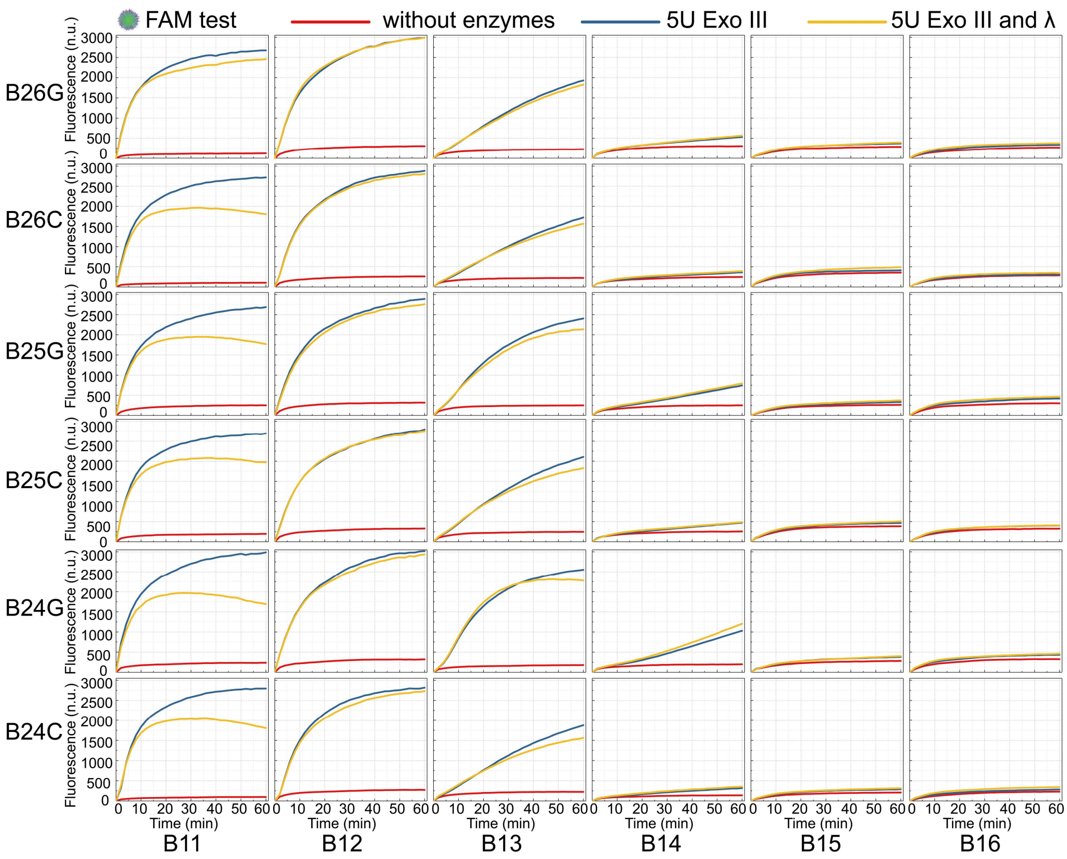

Supplementary Figure 4. FAM curves of the substrate with gap combinations composed of mismatched strands. The hydrolysis of the substrates is tested under different exonucleases backgrounds, which include a control group without enzymes, Exo III, and a coexistence of Exo III and Exo λ. The output signal B1 is monitored by F1.

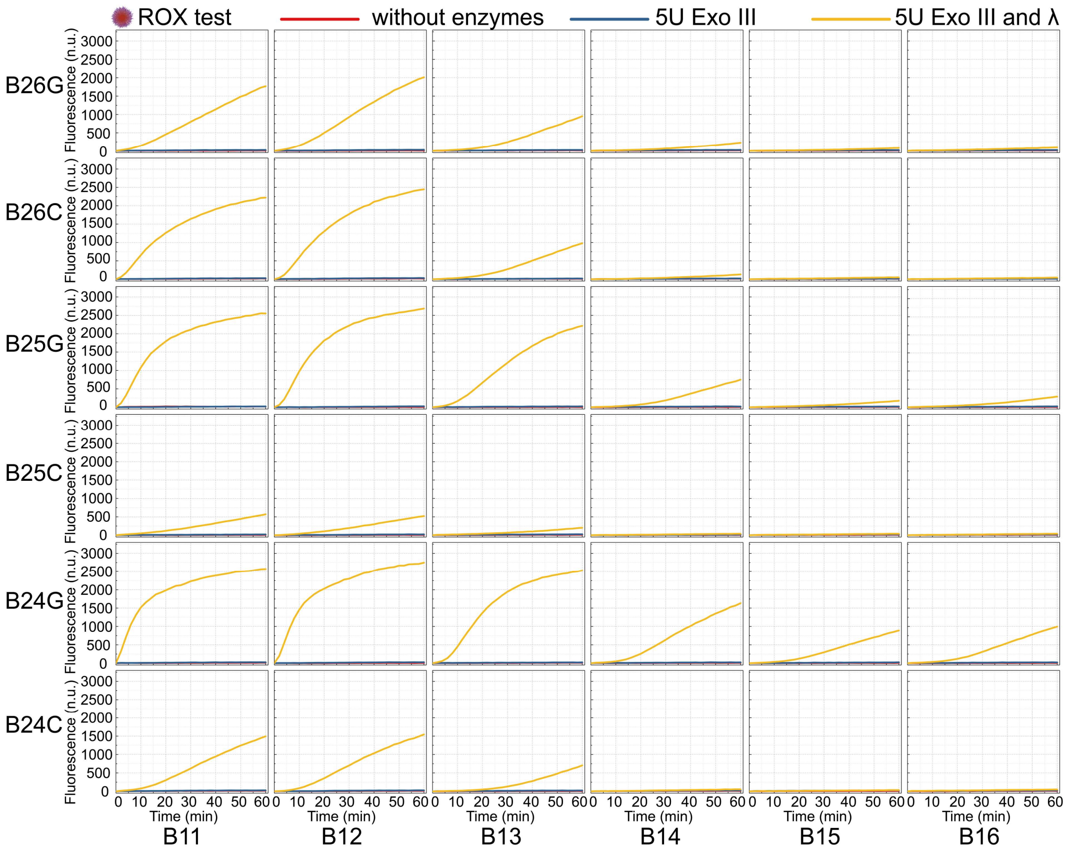

Supplementary Figure 5. ROX curves of the substrate with gap combinations composed of mismatched strands. The hydrolysis of the substrates is tested under different exonucleases backgrounds, which include a control group without enzymes, Exo III, and a coexistence of Exo III and Exo λ. The output signal B1 is monitored by F1.

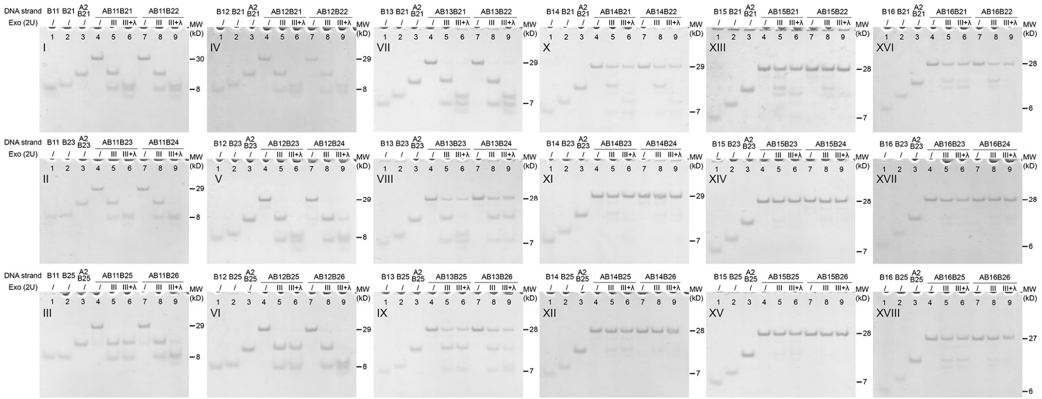

Supplementary Figure 6. PAGE gel matrix for the hydrolysis experiment of the substrate with all gap combinations. The hydrolysis of the substrates with all gap combinations are tested under different exonucleases backgrounds, which include a control group without enzymes, Exo III, and a coexistence of Exo III and Exo λ. The hydrolysis reactions are incubated for 1 h.

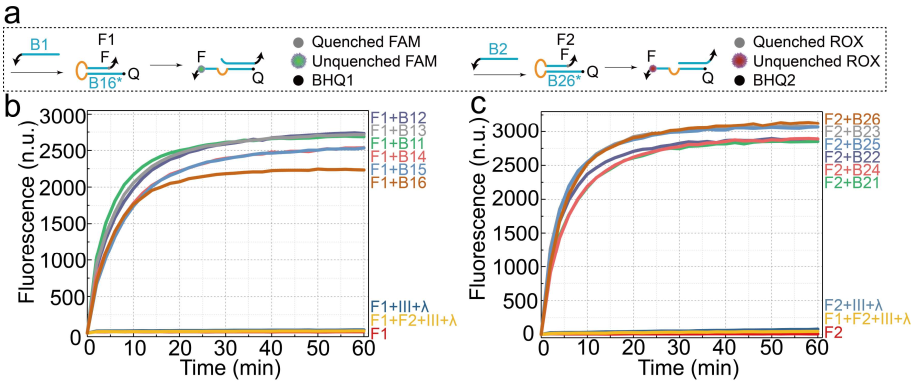

Supplementary Figure 7. Fluorescence measurements of reporters. a, Schematic diagram of the fluorescence produced by F1 and F2. **b,** Fluorescence response of F1. With the addition of B11 to B16, F1 is triggered to produce the positive FAM signal. **c,** Fluorescence response of F2. With the addition of B21 to B26, F2 is triggered to produce the positive ROX signal. The fluorescence curves confirmed that F1 and F2 can only be triggered by their corresponding inputs to generate fluorescence signals without cross-talk or interference.

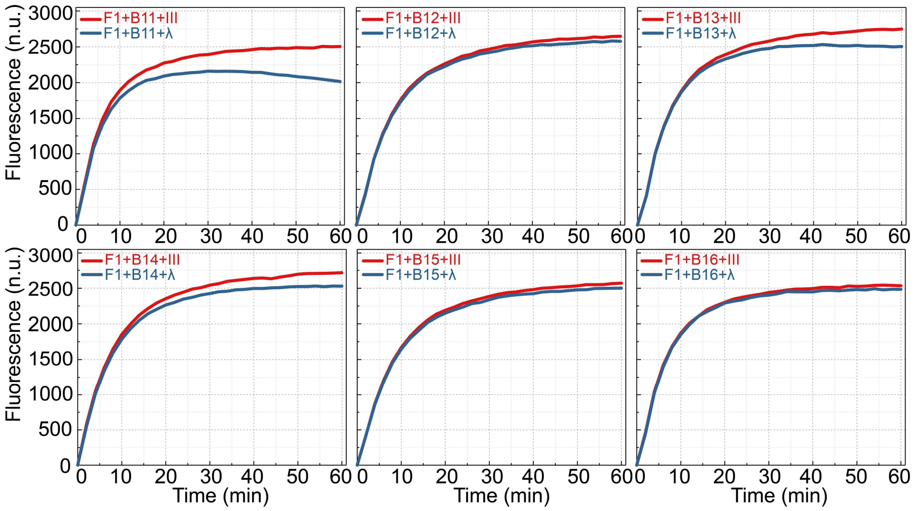

Supplementary Figure 8. FAM curves of hairpin F1 opened by B1 under different exonucleases. The effects of different exonucleases on the opening process of F1 are tested. The fluorescence shows an attenuation in the cases of B11 and B13 with the addition of Exo λ.

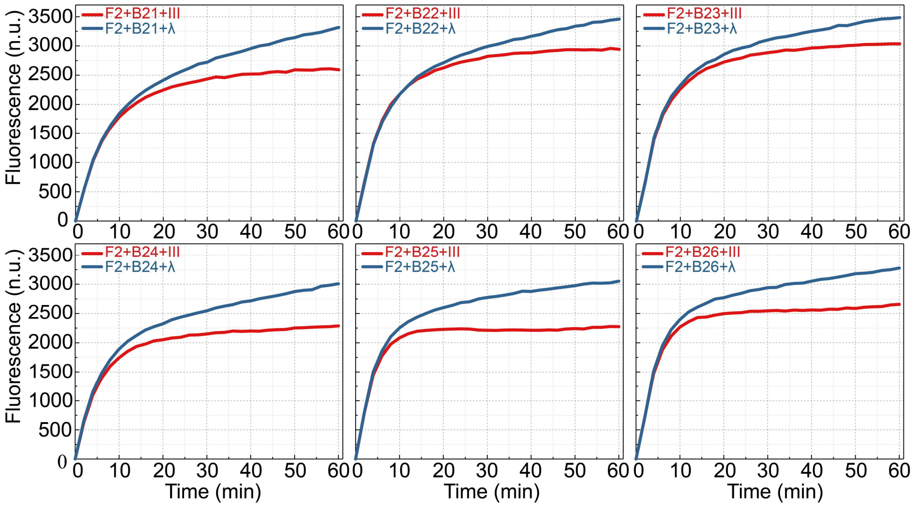

Supplementary Figure 9. ROX curves of hairpin F2 opened by B2 under different exonucleases. The effects of different exonucleases on the opening process of F2 are tested, which shows an increase with the addition of Exo λ, relative to the addition of Exo III.

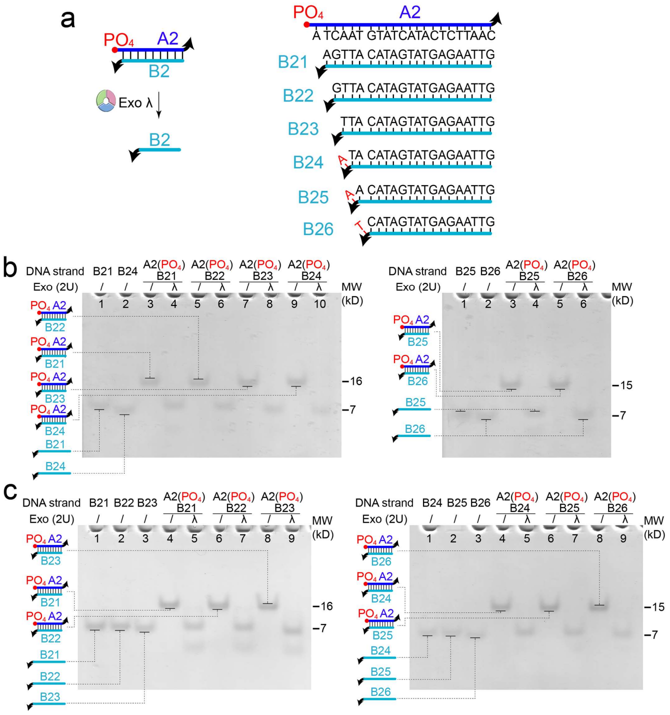

Supplementary Figure 10. Impact of 5' overhang length on Exo λ hydrolysis efficiency. a, Hydrolysis principle of substrates with 5' phosphorylated overhangs. The length variation of B21 to B26 regulates the base number of 5' protruding end of dsDNA. **b,** Hydrolysis assay of substrates with varying 5' overhang lengths in 1 h. **c,** Hydrolysis assay of substrates with varying 5' overhang lengths in 0.5 h. The number of bases in the 5' overhang has almost no effect on the hydrolysis of Exo λ within the designated reaction time.

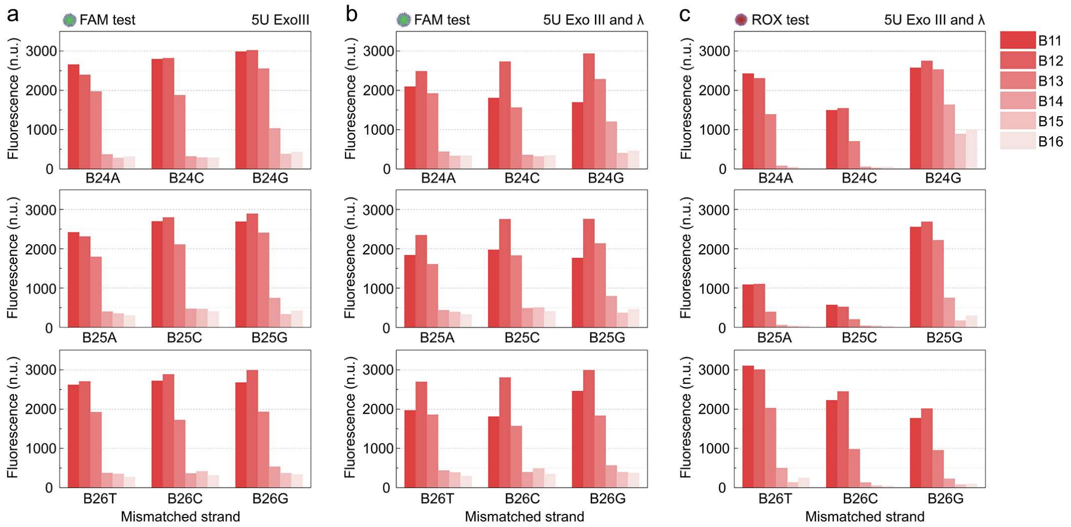

Supplementary Figure 11. Effect of mismatched bases on the hydrolysis of AB1B2.

a, FAM test of substrate hydrolyzed by 5U Exo III. **b,** FAM test of substrate hydrolyzed by 5U Exo III and Exo λ. **c,** ROX test of substrate hydrolyzed by 5U Exo III and Exo λ.

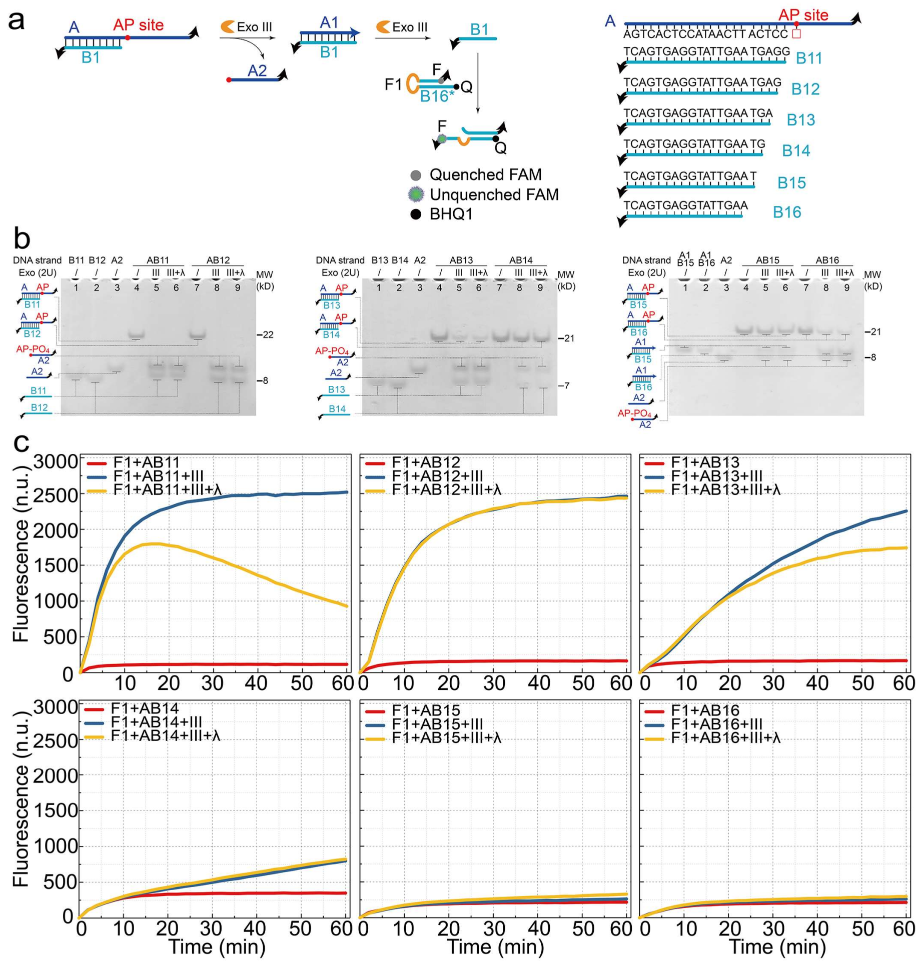

Supplementary Figure 12. Hydrolysis experiment of partial dsDNA with exposed AP site. a, Hydrolysis principle of AB1. The length of B1 ranges from B11 to B16, which is used to modulate the distance between B1 and the AP site. **b,** Gel analysis of hydrolysis experiment for AB1. The hydrolysis of AB11 to AB15 by exonucleases exhibits a decreasing trend. Although the hydrolysis of AB16 is more thorough compared to AB15, it is difficult to generate the output signal B1. **c,** Fluorescence analysis of hydrolysis experiment for AB1. Fluorescence attenuation is observed in the case of AB11 during the cooperative hydrolysis driven by Exo III and Exo λ, which is consistent with the case of B11 triggering F1 in Supplementary Figure 8.

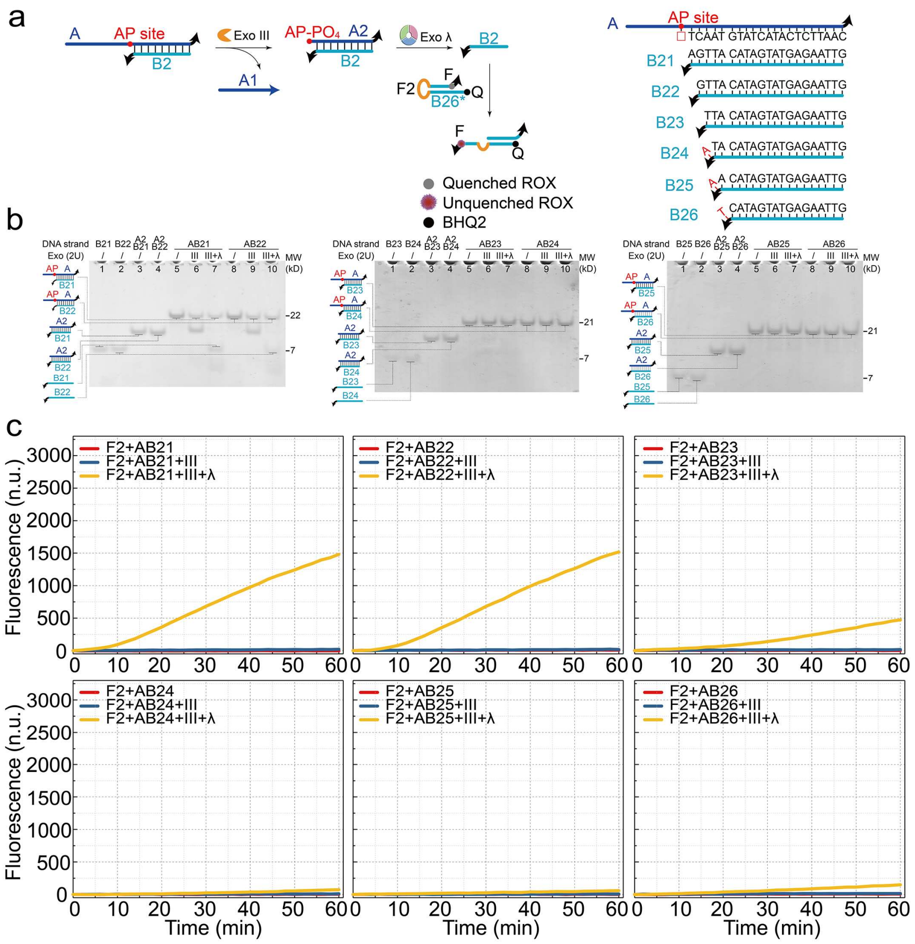

Supplementary Figure 13. Hydrolysis experiment of partial dsDNA with exposed AP site. a, Hydrolysis principle of AB2. The length of B2 ranges from B21 to B26, which is used to modulate the distance between B2 and the AP site. **b,** Gel analysis of hydrolysis experiment for AB2. AB21 to AB26 are unable to be completely consumed during the 1 h incubation, and there is an increasing trend in the residual substrates. **c,** Fluorescence analysis of hydrolysis experiment for AB2. The output signal shows attenuation as the substrates varies from AB21 to AB26, and during the transition of AB24 to AB26, there is no effective output.

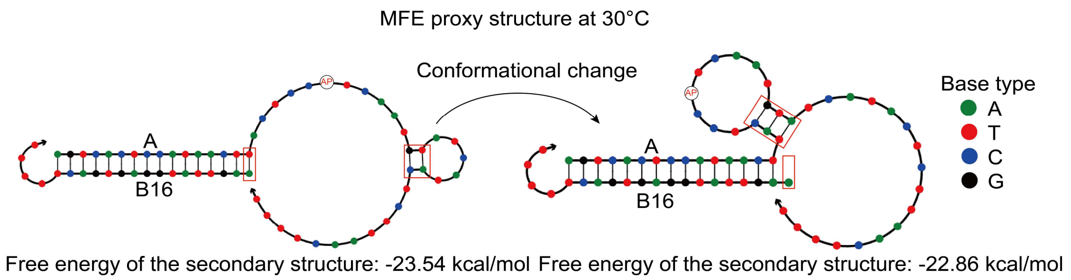

Supplementary Figure 14. NUPACK Simulation for the conformational change of AB16. Due to the relative instability of A-T base pairs compared to G-C base pairs, especially when located at the ends of dsDNA, it is possible for A to dissociate from T. To investigate the conformational changes of AB16 after the dissociation of A and T at the dsDNA end, structure simulations are performed to reveal the conformational changes of AB16 and the secondary structure formed near the AP site, which may be one of the causes leading to erroneous hydrolysis at the AP site.

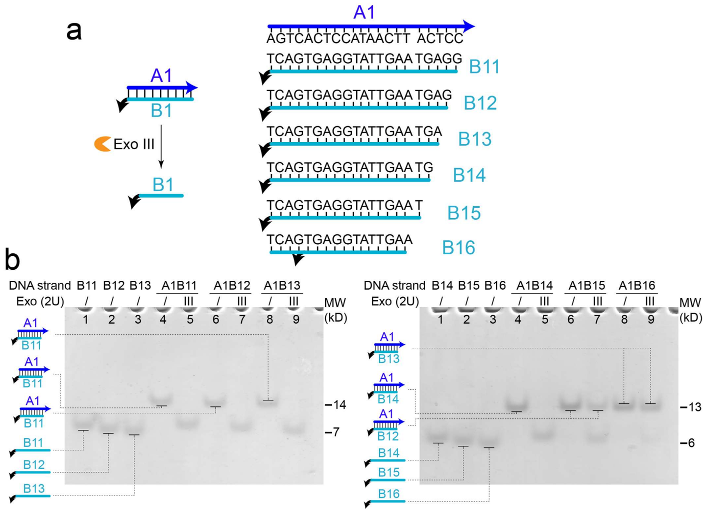

Supplementary Figure 15. Impact of 3' overhang length on Exo III hydrolysis efficiency. a, Hydrolysis principle of substrates with 3' overhangs. The length variation of B11 to B16 regulates the base number of 3' protruding end of dsDNA. **b,** Hydrolysis assay of substrates with varying 3' overhang lengths in 1 h. The hydrolysis of Exo III on the substrate decreases as the number of 3' overhang bases increases.

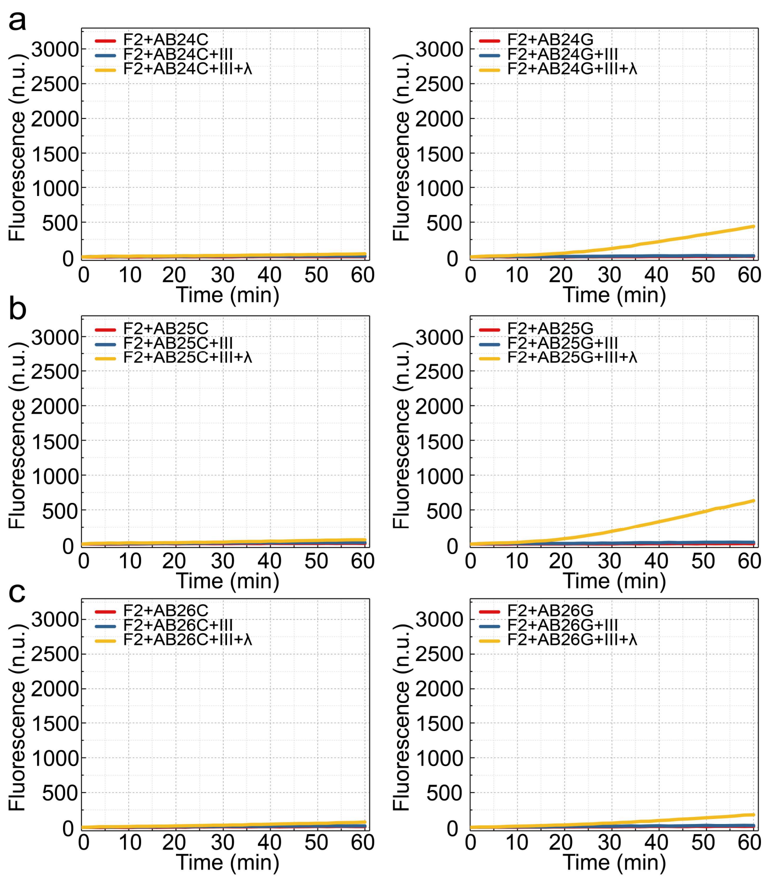

Supplementary Figure 16. ROX curves of hydrolysis experiment for AB2 with mismatched bases. a, Fluorescence analysis of AB24 with mismatched bases. b, Fluorescence analysis of AB25 with mismatched bases. c, Fluorescence analysis of AB26 with mismatched bases.

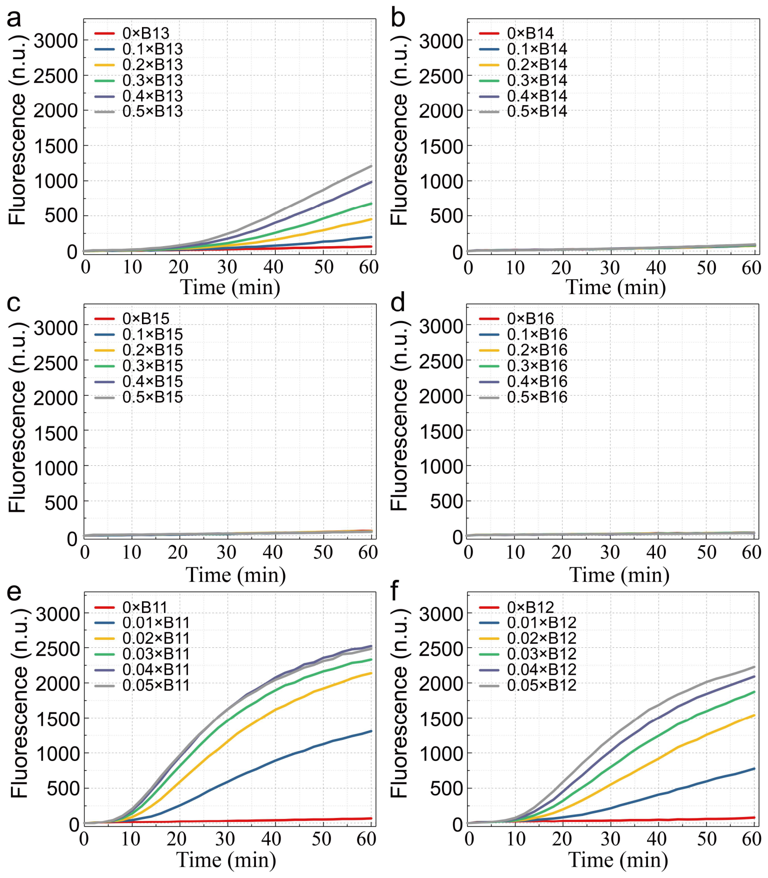

Supplementary Figure 17. Fluorescence analysis of catalytic reaction without DSD. The fluorescence of catalytic reaction is monitored with the input concentrations of B13 **(a)**, B14 **(b)**, B15 **(c)**, and B16 **(d)** ranging from 0.1× to 0.5× 0.2 μM, and the input concentrations of B11 **(e)**, B12 **(f)** ranging from 0.01× to 0.05× 0.2 μM.

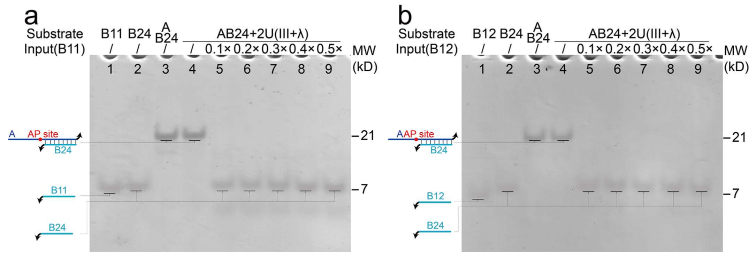

Supplementary Figure 18. Gel analysis of catalytic reaction without DSD. a, Gel verification of catalytic reaction with B11 as input. **b,** Gel verification of catalytic reaction with B12 as input. Both gels confirms that AB24 can be completely consumed under the catalytic reaction, resulting in the output signal without the generation of byproducts that could affect the reaction.

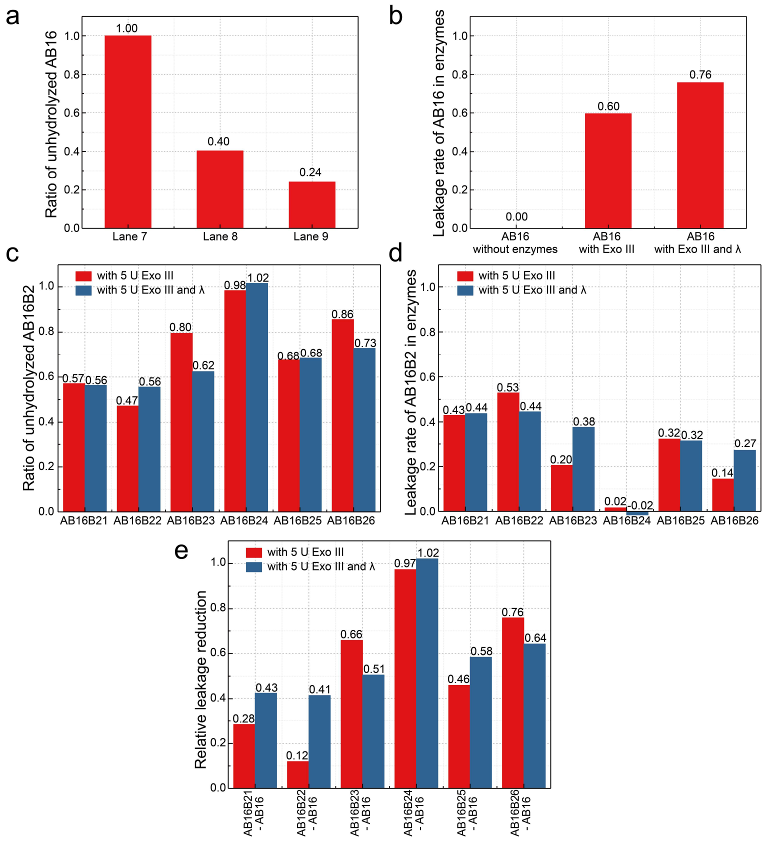

Supplementary Figure 19. Quantitative analysis of leakage using software of Image J. a, The analysis of unhydrolyzed AB16 in Supplementary Fig. 12c by software Image J. The grayscale value of lane 7 is measured as standard value by software Image J, then, the ratio of unhydrolyzed AB16 in lanes 8 and 9 are calculated as the measured grayscale value of lanes 8 and 9 divided by the grayscale value of lane 7, respectively. **b,** The leakage rate of AB16 with enzymes in Supplementary Fig. 19a. The rate of hydrolyzed AB16 is calculated as 1 minus the rate of unhydrolyzed AB16, which is the leakage rate of AB16. **c,** The analysis of unhydrolyzed AB16B2 with enzymes in XVI-XVIII of Supplementary Fig. 6 by software Image J. The grayscale value of AB16B2 without enzymes is measured as standard value by software Image J. The ratio of unhydrolyzed AB16B2 with enzymes is calculated as the measured grayscale value of AB16B2 with enzymes divided by the grayscale value of AB16B2 without enzymes. **d,** The leakage rate of AB16B2 with enzymes in XVI-XVIII of Supplementary Fig. 6. The rate of hydrolyzed AB16B2 with enzymes is calculated as 1 minus the rate of unhydrolyzed AB16B2 with enzymes, which is the leakage rate of AB16B2. **e,** The leakage reduction ratio of AB16B2 relative to AB16.

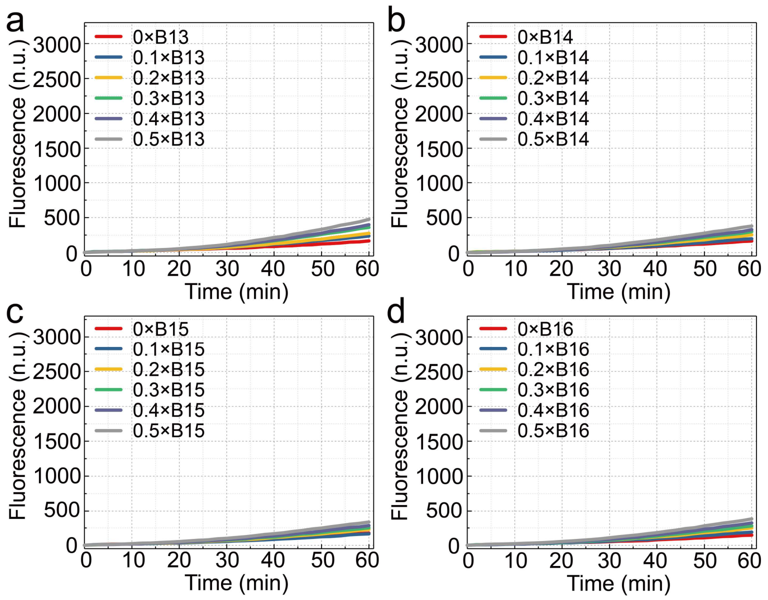

Supplementary Figure 20. Fluorescence analysis of catalytic reaction with DSD. The fluorescence of catalytic reaction is monitored with the input concentrations of B13 **(a)**, B14 **(b)**, B15 **(c)**, and B16 **(d)** ranging from 0.1× to 0.5× 0.2 μM.

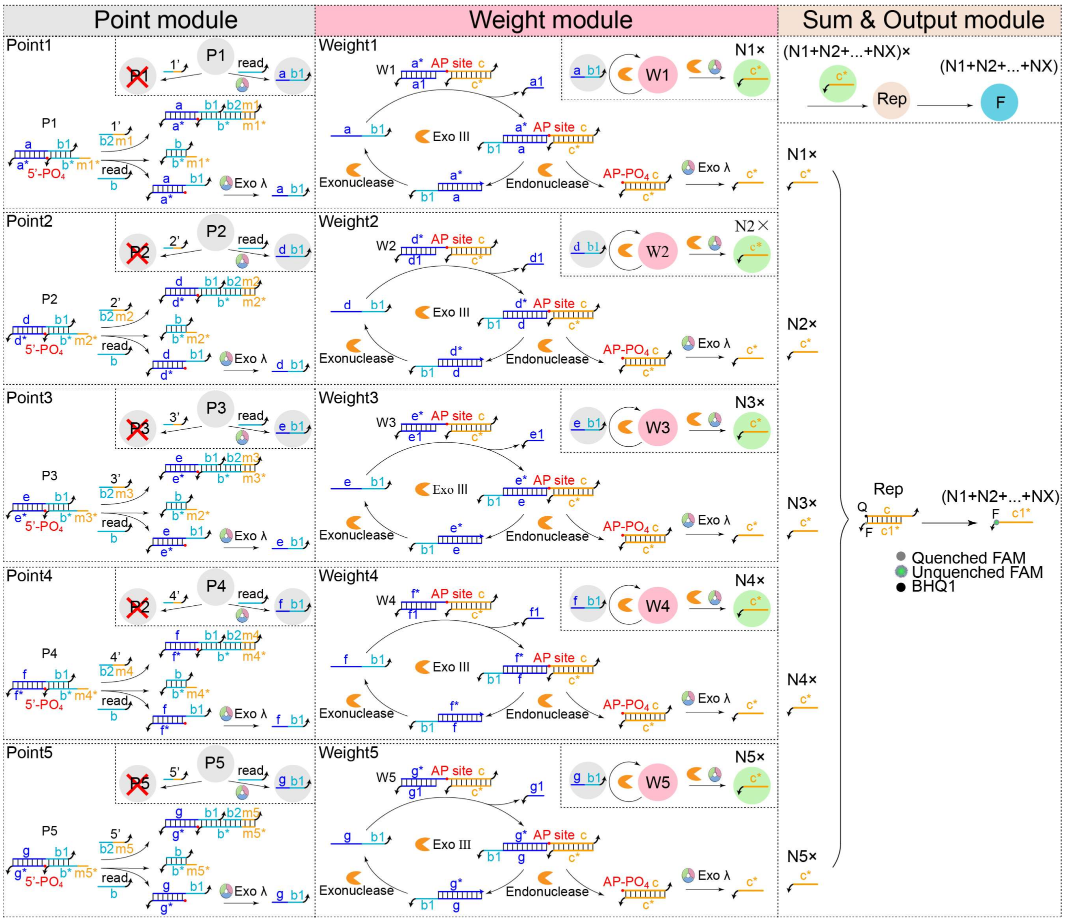

Supplementary Figure 21. Molecular computing model for solving the MWCP. The model consists of three kinds of modules: the Point module, Weight module, and Sum & Output module. The reactions of these three modules can be performed in parallel between vertices. Ultimately, the numerical data is transformed by a unified FAM fluorescence signal.

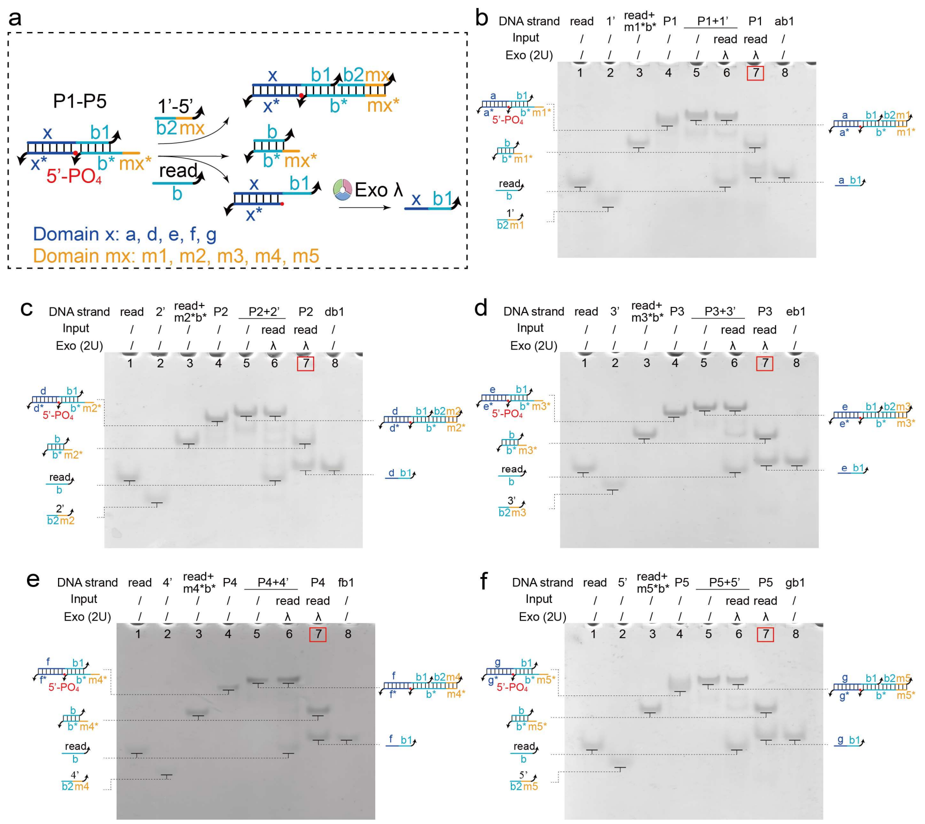

Supplementary Figure 22. PAGE analysis of Point module inhibition and activation.

a, Schematic diagram of Point module inhibition and activation. PAGE analysis of Point1 **(b)**, Point2 **(c)**, Point3 **(d)**, Point4 **(e)** and Point5 **(f)**. All vertices can be activated and triggered to generate the output signal under the condition of non-inhibition by a common input.

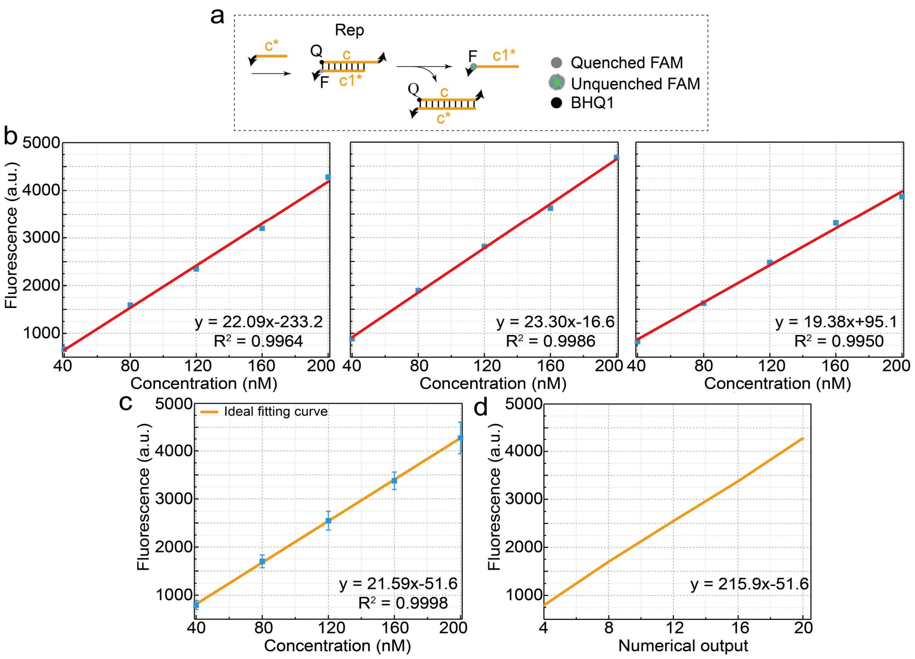

Supplementary Figure 23. Quantitative analysis of Rep triggered by gradient input signals. a, Schematic diagram of ideal fluorescence signal. **b,** Fluorescence signals triggered by gradient concentration of input. Three identical experiments are performed with the constant concentration of 200 nM for Rep. **c,** Linear fitting of the averaged output signal corresponding to the gradient concentration of input. The linear function between the fluorescence signal and the input concentrations, as well as the correlation coefficient (R^2) for the fitted line, are calculated. The averaged fluorescence is considered as the idealized output signal. The error bars represent the standard deviation of three sets of data (n=3 independent experiments). **d,** The correspondence between numerical output and fluorescence value. The fluorescence data of the fitted line is converted into numerical values representing the output.

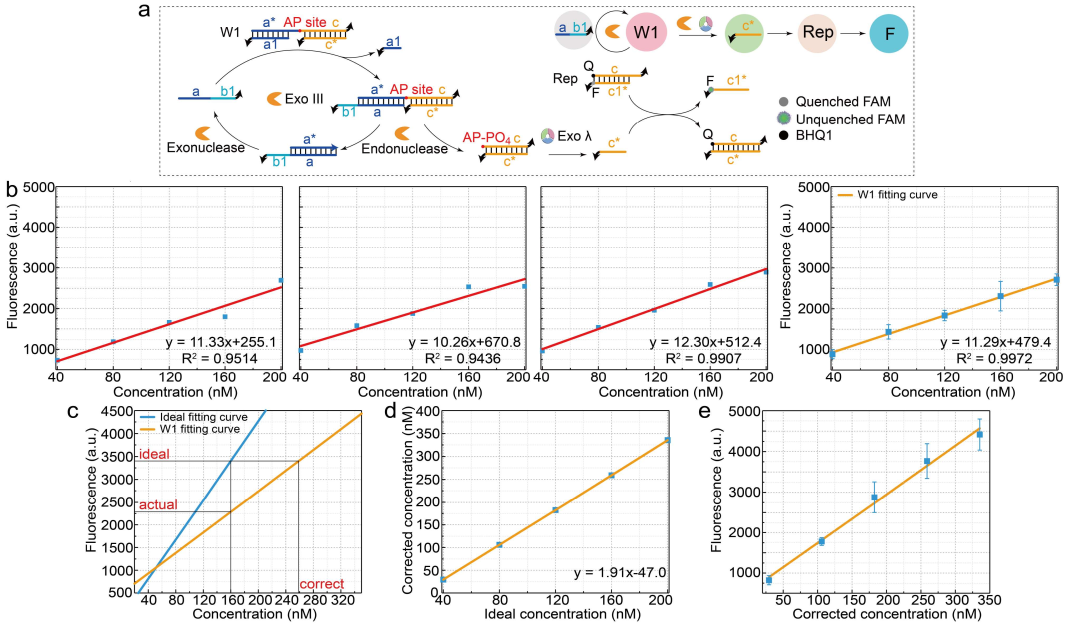

Supplementary Figure 24. Fluorescent intensity regulated by substrate concentration of weight1 module. a, Schematic diagram of the Weight1 module cascaded with the Sum & Output module. **b,** The actual fluorescence response curve of the substrate in the Weight1 module. With a constant concentration of 200 nM for Rep, the concentrations of W1 are adjusted based on ideal weights to obtain the actual fluorescence response curve of the Weight1 module, followed by linear fitting and the calculation of the correlation coefficient. **c,** Substrate concentration of Weight1 for the ideal fluorescence. Taking the substrate concentration of 160 nM for the Weight1 module as an example, the fluorescence triggered is measured as the actual value, which is difficult to achieve the ideal fluorescence intensity. Therefore, it is necessary to adjust the concentration of the W1 to obtain the ideal fluorescence and meet the final output requirements. **d,** The calibration for the substrate concentration of Weight1 to achieve the ideal fluorescence. The concentrations of the W1 are adjusted according to the ideal fluorescence. **e,** Fluorescence response for the corrected substrate concentration of the Weight1 module. The error bars represent the standard deviation of three sets of data (n=3 independent experiments).

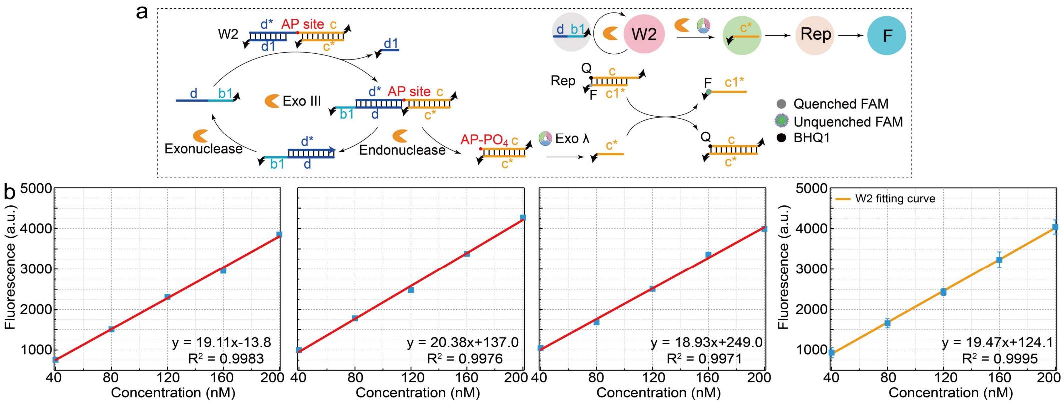

Supplementary Figure 25. Fluorescent intensity regulated by substrate concentration of weight2 module. a, Schematic diagram of the Weight2 module cascaded with the Sum & Output module. **b,** The actual fluorescence response curve of the substrate in the Weight2 module. With a constant concentration of 200 nM for Rep, the concentrations of W2 are adjusted based on ideal weights to obtain the actual fluorescence response curve of the Weight2 module, followed by linear fitting and calculation of the correlation coefficient. The variance in fluorescence between the ideal curve (Supplementary Figure 23c) and the actual curve (the right panel) at the same substrate concentration ranges within the interval $[-175.7, 205.9]$, $(x \in [0, 180])$, which is less pronounced compared to the slope in Supplementary Figure 23d, and the range of concentrations can meet the setting for vertex 2. The slope signifies the rate of change in fluorescence intensity for each unit change in numerical output. The error bars represent the standard deviation of three sets of data (n=3 independent experiments).

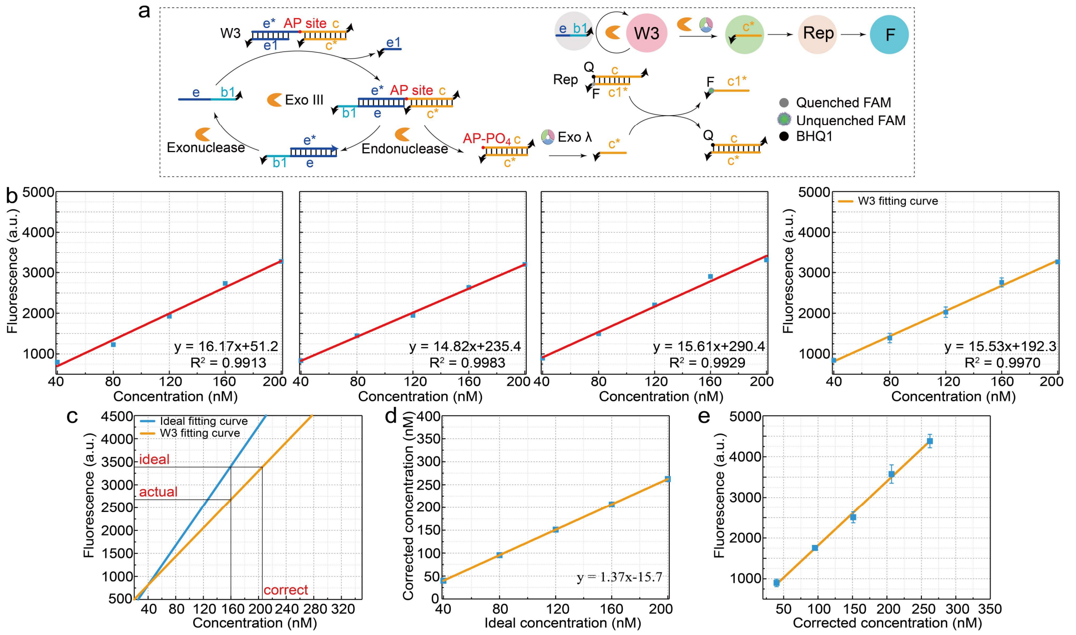

Supplementary Figure 26. Fluorescent intensity regulated by substrate concentration of weight3 module. a, Schematic diagram of the Weight3 module cascaded with the Sum & Output module. **b,** The actual fluorescence response curve of the substrate in the Weight3 module. With a constant concentration of 200 nM for Rep, the concentrations of W3 are adjusted based on ideal weights to obtain the actual fluorescence response curve of the Weight3 module, followed by linear fitting and the calculation of the correlation coefficient. **c,** Substrate concentration of Weight3 for the ideal fluorescence. The method to adjust the concentration of the W3 is consistent with the Weight1 module. **d,** The calibration for the substrate concentration of Weight3 to achieve the ideal fluorescence. **e,** Fluorescence response for the corrected substrate concentration of the Weight3 module. The error bars represent the standard deviation of three sets of data (n=3 independent experiments).

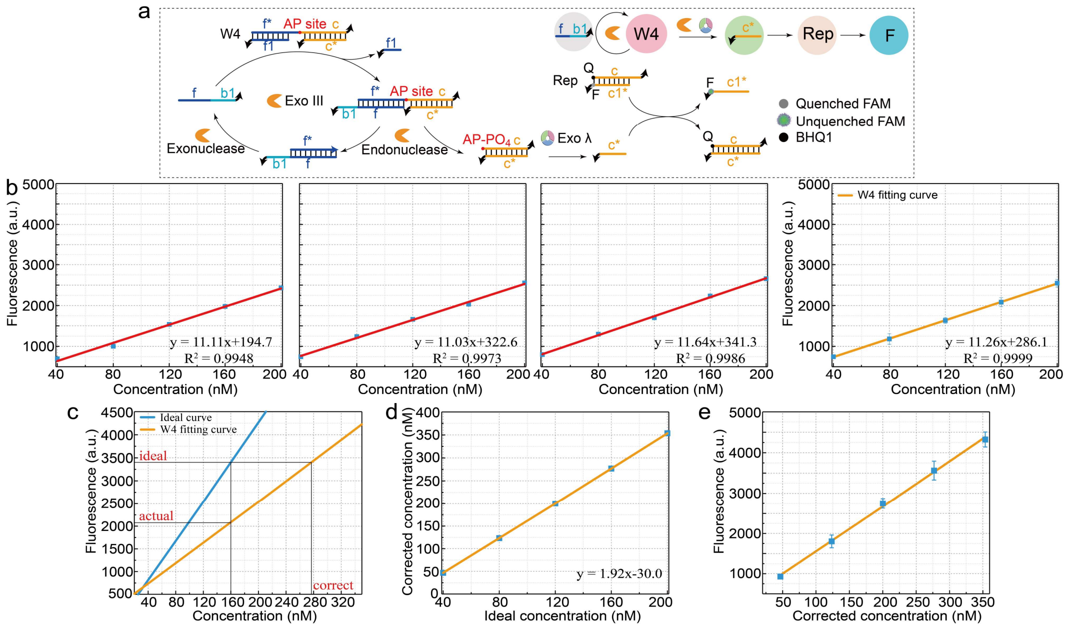

Supplementary Figure 27. Fluorescent intensity regulated by substrate concentration of weight4 module. a, Schematic diagram of the Weight4 module cascaded with the Sum & Output module. **b,** The actual fluorescence response curve of the substrate in the Weight4 module. With a constant concentration of 200 nM for Rep, the concentrations of W4 are adjusted based on ideal weights to obtain the actual fluorescence response curve of the Weight4 module, followed by linear fitting and the calculation of the correlation coefficient. **c,** Substrate concentration of Weight4 for the ideal fluorescence. The method to adjust the concentration of the W4 is consistent with the Weight1 module. **d,** The calibration for the substrate concentration of Weight4 to achieve the ideal fluorescence. **e,** Fluorescence response for the corrected substrate concentration of the Weight4 module. The error bars represent the standard deviation of three sets of data (n=3 independent experiments).

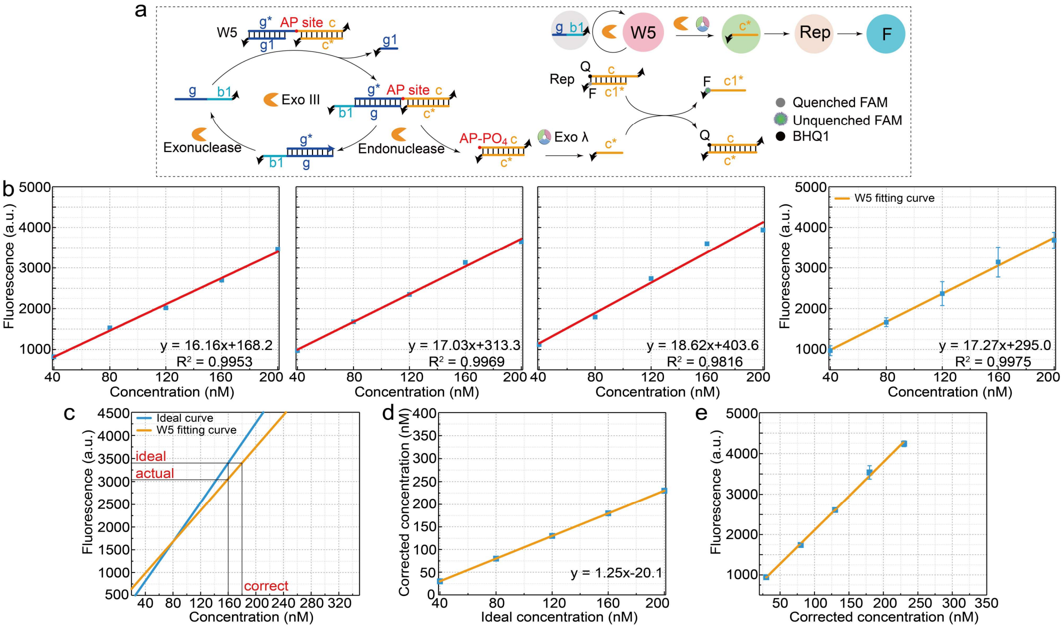

Supplementary Figure 28. Fluorescent intensity regulated by substrate concentration of weight5 module. a, Schematic diagram of the Weight5 module cascaded with the Sum & Output module. **b,** The actual fluorescence response curve of the substrate in the Weight5 module. With a constant concentration of 200 nM for Rep, the concentrations of W5 are adjusted based on ideal weights to obtain the actual fluorescence response curve of the Weight5 module, followed by linear fitting and the calculation of the correlation coefficient. **c,** Substrate concentration of Weight5 for the ideal fluorescence. The method to adjust the concentration of the W5 is consistent with the Weight1 module. **d,** The calibration for the substrate concentration of Weight5 to achieve the ideal fluorescence. **e,** Fluorescence response for the corrected substrate concentration of the Weight5 module. The error bars represent the standard deviation of three sets of data (n=3 independent experiments).

Uncropped and unedited blotgel images

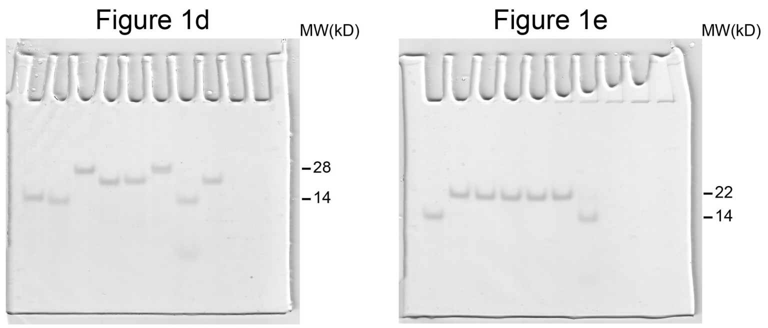

Supplementary Figure 29. Gels corresponding to Figures 1d and 1e.

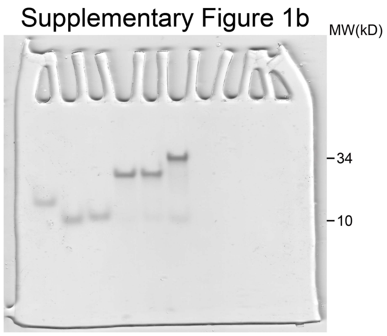

Supplementary Figure 30. Gel corresponding to Supplementary Figure 1b.

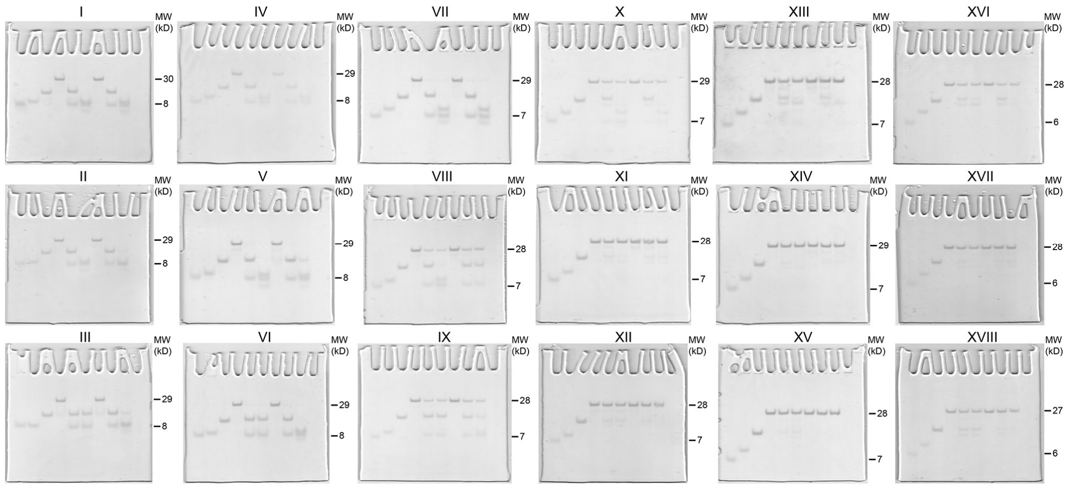

Supplementary Figure 31. Gels corresponding to Supplementary Figure 6.

Supplementary Figure 10b

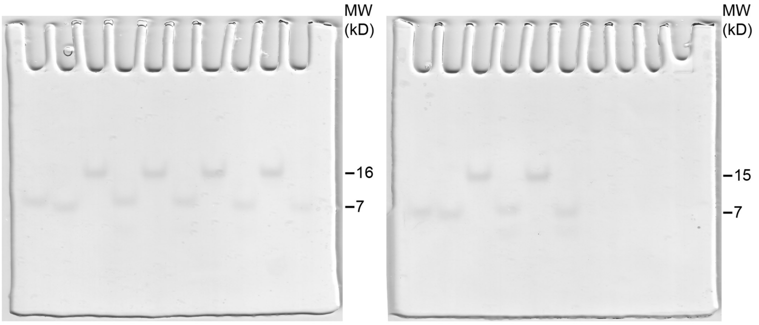

Supplementary Figure 10c

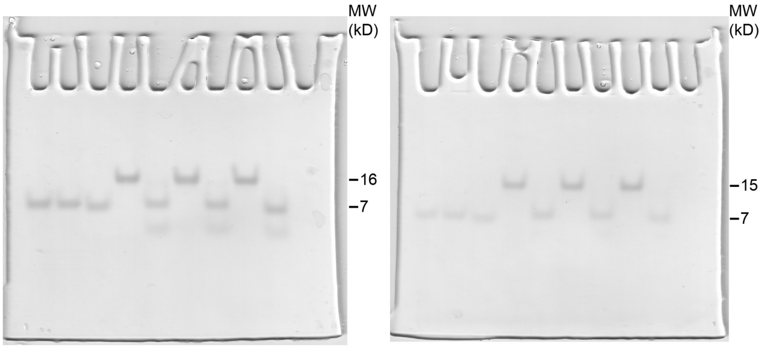

Supplementary Figure 32. Gels corresponding to Supplementary Figures 10b and 10c.

Supplementary Figure 12b

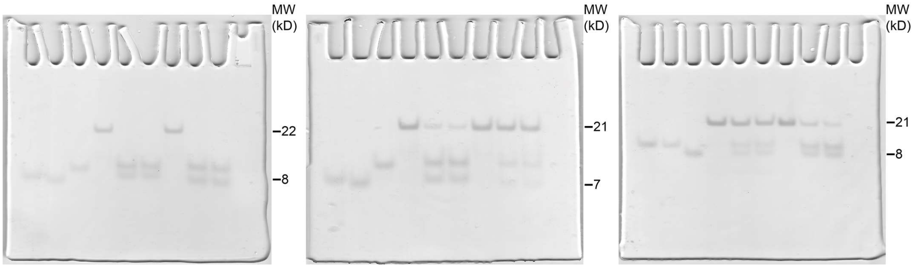

Supplementary Figure 33. Gels corresponding to Supplementary Figure 12b.

Supplementary Figure 13b

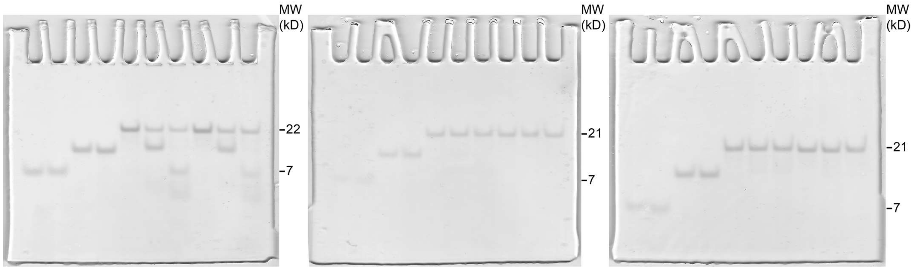

Supplementary Figure 34. Gels corresponding to Supplementary Figure 13b.

Supplementary Figure 15b

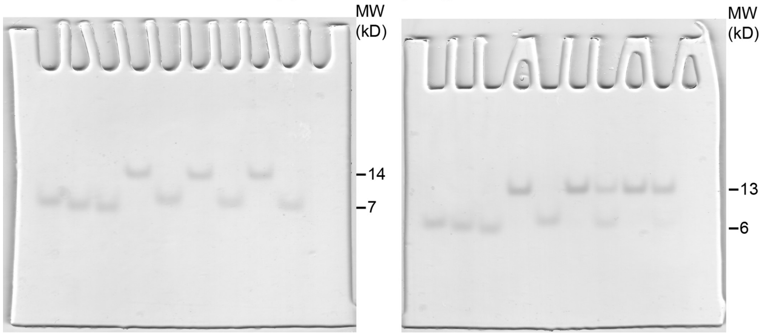

Supplementary Figure 35. Gels corresponding to Supplementary Figure 15b.

Supplementary Figure 18a

Supplementary Figure 18b

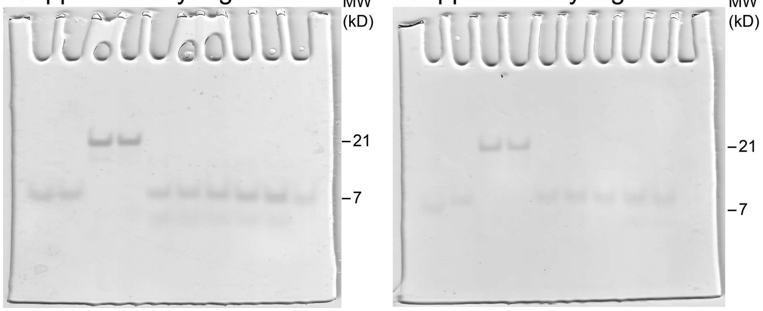

Supplementary Figure 36. Gels corresponding to Supplementary Figures 18a and 18b.

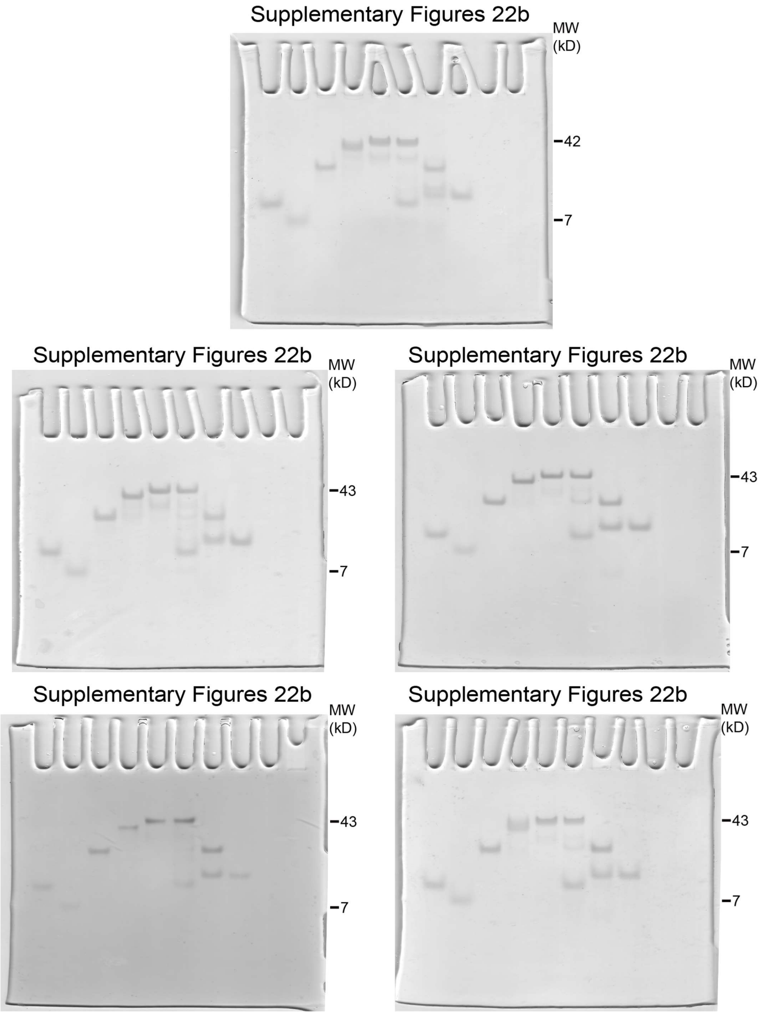

Supplementary Figure 37. Gels corresponding to Supplementary Figures 22b-22f.
