# Supplementary material for: A signal transmission strategy driven by gap-regulated exonuclease hydrolysis for hierarchical molecular networks
Source: Commun Biol. 2024 Mar 16;7:335. doi: 10.1038/s42003-024-06036-5 (PMC10944543; doi:10.1038/s42003-024-06036-5)
Supplement: Supplementary file 3 — Description of Additional Supplementary Files [file 42003_2024_6036_MOESM3_ESM.pdf]

Description of Additional Supplementary Files

File name: Supplementary Data 1
Description: The source data of fluorescence behind the graphs in the article.

File name: Supplementary Data 2
Description: The source data of fluorescence behind the graphs in the Supplementary Information file.
