# Supplementary material for: A signal transmission strategy driven by gap-regulated exonuclease hydrolysis for hierarchical molecular networks
Source: Commun Biol. 2024 Mar 16;7:335. doi: 10.1038/s42003-024-06036-5 (PMC10944543; doi:10.1038/s42003-024-06036-5)
Supplement: Supplementary file 6 — Reporting Summary [file 42003_2024_6036_MOESM6_ESM.pdf]

nature portfolio

Corresponding author(s): Qaing Zhang

Last updated by author(s): Feb 21, 2024

Reporting Summary

Nature Portfolio wishes to improve the reproducibility of the work that we publish. This form provides structure for consistency and transparency in reporting. For further information on Nature Portfolio policies, see our Editorial Policies and the Editorial Policy Checklist.

Statistics

For all statistical analyses, confirm that the following items are present in the figure legend, table legend, main text, or Methods section.

n/a	Confirmed	
☐	☒	The exact sample size (*n*) for each experimental group/condition, given as a discrete number and unit of measurement
☐	☒	A statement on whether measurements were taken from distinct samples or whether the same sample was measured repeatedly
☒	☐	The statistical test(s) used AND whether they are one- or two-sided<br>*Only common tests should be described solely by name; describe more complex techniques in the Methods section.*
☐	☒	A description of all covariates tested
☐	☒	A description of any assumptions or corrections, such as tests of normality and adjustment for multiple comparisons
☐	☒	A full description of the statistical parameters including central tendency (e.g. means) or other basic estimates (e.g. regression coefficient) AND variation (e.g. standard deviation) or associated estimates of uncertainty (e.g. confidence intervals)
☒	☐	For null hypothesis testing, the test statistic (e.g. *F*, *t*, *r*) with confidence intervals, effect sizes, degrees of freedom and *P* value noted<br>*Give P values as exact values whenever suitable.*
☒	☐	For Bayesian analysis, information on the choice of priors and Markov chain Monte Carlo settings
☐	☒	For hierarchical and complex designs, identification of the appropriate level for tests and full reporting of outcomes
☒	☐	Estimates of effect sizes (e.g. Cohen's *d*, Pearson's *r*), indicating how they were calculated

Our web collection on statistics for biologists contains articles on many of the points above.

Software and code

Policy information about availability of computer code

Data collection	SPARKCONTROL Dashboard 1.0.0.0
Data analysis	Microsoft Office 2021, OriginPro 2017

For manuscripts utilizing custom algorithms or software that are central to the research but not yet described in published literature, software must be made available to editors and reviewers. We strongly encourage code deposition in a community repository (e.g. GitHub). See the Nature Portfolio guidelines for submitting code & software for further information.

Data

Policy information about availability of data

All manuscripts must include a data availability statement. This statement should provide the following information, where applicable:

- Accession codes, unique identifiers, or web links for publicly available datasets
- A description of any restrictions on data availability
- For clinical datasets or third party data, please ensure that the statement adheres to our policy

The source data for all figures of fluorescence is available in Supplementary Data 1 and 2. All figures are open on figshare (https://figshare.com/s/3c62963ac3e5ee2521c8). Uncropped gels are included in the Supplementary Figures. 29-37. Any further data not included in the text will be made available upon request.

Research involving human participants, their data, or biological material

Policy information about studies with human participants or human data. See also policy information about sex, gender (identity/presentation), and sexual orientation and race, ethnicity and racism.

Reporting on sex and gender	This article does not involve the sex- and gender-based research.
Reporting on race, ethnicity, or other socially relevant groupings	This article does not involve the study of race, ethnicity, or other socially relevant groups.
Population characteristics	This article does not involve human research participants.
Recruitment	This article does not require recruitment of any human researchers.
Ethics oversight	The National Natural Science Foundation of China, Natural Science Foundation of Liaoning Province, the Postgraduate Education Reform Project of Liaoning Province, and the Dalian Outstanding Young Science and Technology Talent Support Program.

Note that full information on the approval of the study protocol must also be provided in the manuscript.

Field-specific reporting

Please select the one below that is the best fit for your research. If you are not sure, read the appropriate sections before making your selection.

☒ Life sciences ☐ Behavioural & social sciences ☐ Ecological, evolutionary & environmental sciences

For a reference copy of the document with all sections, see nature.com/documents/nr-reporting-summary-flat.pdf

Life sciences study design

All studies must disclose on these points even when the disclosure is negative.

Sample size	In order to determine the average value and standard deviation of each data point to complete the drawing of curves/bar charts and their error bars, this article sets up three independent repeated experiments for each data point to collect data.
Data exclusions	No data were excluded from the analyses.
Replication	All repeatability in the experiment has been verified, but the effect may be affected by enzyme activity, environmental conditions, etc. Especially for the enzyme activity, the activity of newly purchased Exo lambda will decay to a relatively stable state after starting use, which will affect the reaction rate of the experiment, but will not affect the results.
Randomization	The control group has only a single variable to obtain reliable experimental results, which is a representative and evenly distributed value within a range to ensure a sufficient explanation of the experimental phenomenon.
Blinding	Due to the independent testing of repeated experiments in this article, and the inability to influence each other, blinding is not relevant to our study.

Reporting for specific materials, systems and methods

We require information from authors about some types of materials, experimental systems and methods used in many studies. Here, indicate whether each material, system or method listed is relevant to your study. If you are not sure if a list item applies to your research, read the appropriate section before selecting a response.

Materials & experimental systems

n/a	Involved in the study	
☒	☐	Antibodies
☒	☐	Eukaryotic cell lines
☒	☐	Palaeontology and archaeology
☒	☐	Animals and other organisms
☒	☐	Clinical data
☐	☒	Dual use research of concern
☒	☐	Plants

Methods

n/a	Involved in the study	
☒	☐	ChIP-seq
☒	☐	Flow cytometry
☒	☐	MRI-based neuroimaging

Dual use research of concern

Policy information about [dual use research of concern]

Hazards

Could the accidental, deliberate or reckless misuse of agents or technologies generated in the work, or the application of information presented in the manuscript, pose a threat to:

No	Yes	
☒	☐	Public health
☒	☐	National security
☒	☐	Crops and/or livestock
☒	☐	Ecosystems
☒	☐	Any other significant area

Experiments of concern

Does the work involve any of these experiments of concern:

No	Yes	
☒	☐	Demonstrate how to render a vaccine ineffective
☒	☐	Confer resistance to therapeutically useful antibiotics or antiviral agents
☒	☐	Enhance the virulence of a pathogen or render a nonpathogen virulent
☒	☐	Increase transmissibility of a pathogen
☒	☐	Alter the host range of a pathogen
☒	☐	Enable evasion of diagnostic/detection modalities
☒	☐	Enable the weaponization of a biological agent or toxin
☒	☐	Any other potentially harmful combination of experiments and agents

Plants

Seed stocks	This article does not involve the study of plants or seeds.
Novel plant genotypes	See above
Authentication	See above
